# Risankizumab for the treatment of moderate‐to‐severe psoriasis: A multicenter, retrospective, 1 year real‐life study

**DOI:** 10.1111/dth.15489

**Published:** 2022-04-13

**Authors:** Giacomo Caldarola, Arianna Zangrilli, Nicoletta Bernardini, Mauro Bavetta, Clara De Simone, Dario Graceffa, Claudio Bonifati, Sara Faleri, Domenico Giordano, Marco Mariani, Adriana Micheli, Gaia Moretta, Gianluca Pagnanelli, Vincenzo Panasiti, Alessia Provini, Antonio Richetta, Ketty Peris, Luca Bianchi

**Affiliations:** ^1^ Dermatologia, Dipartimento di Medicina e Chirurgia Traslazionale Università Cattolica del Sacro Cuore Rome; ^2^ UOC di Dermatologia, Dipartimento di Scienze Mediche e Chirurgiche Fondazione Policlinico Universitario A. Gemelli – IRCCS Rome; ^3^ Department of Dermatology University of Rome Tor Vergata Rome Italy; ^4^ Department of Medical‐Surgical Sciences and Biotechnologies, Dermatology Unit "Daniele Innocenzi" Sapienza University of Rome Rome Italy; ^5^ Istituto Dermatologico San Gallicano – IRCCS Rome Italy; ^6^ UOC DERMATOLOGIA dell'Ospedale di Belcolle Viterbo Italy; ^7^ NESMOS department, dermatology unit, Sant'Andrea hospital University of Rome Sapienza Rome Italy; ^8^ Section of Hygiene, University Department of Health Sciences and Public Health Università Cattolica del Sacro Cuore Rome Italy; ^9^ Unità di Dermatologia, Ospedale Nuovo Regina Margherita Rome Italy; ^10^ Istituto Dermopatico dell'Immacolata – IRCCS Rome Italy; ^11^ Plastic and Reconstructive Surgery Unit Campus Bio‐Medico University Rome Italy; ^12^ Unit of Dermatology, Department of Internal Medicine and Medical Specialties Sapienza University of Rome Rome Italy

**Keywords:** efficacy, interleukin‐23, psoriasis, real‐life, risankizumab, safety

## Abstract

Several new biologic agents targeting IL23/Th17 axis, such as risankizumab, have been developed for the treatment of psoriasis. The aim of the present study was to analyze the efficacy and safety of risankizumab in patients with moderate‐to‐severe psoriasis over a 52‐week period. A multicentric retrospective study was conducted in patients who initiated risankizumab between July 2019 and December 2020. Psoriasis Area and Severity Index—PASI was measured at baseline and after 4, 16, 28 and 52 weeks. Clinical responses were evaluated by PASI75, PASI90 and PASI100 at the same timepoints. Potential safety issues and adverse events (AEs) were collected. Univariable and multivariable logistic regressions were performed for variables predicting clinical response. One hundred and twelve patients with psoriasis were included. PASI90 response was achieved by 17.86% of patients at week 4, 72.22% at week 16, 91.0% at week 28 and 95.24% at week 52 (as observed analysis). No associations between the considered variables and the efficacy endpoints were retrieved, influence of variables such as Body Mass Index (BMI), baseline PASI or previous biologics were not shown. No serious safety issues or discontinuations related to adverse events were reported. Risankizumab showed high efficacy and a favorable safety profile, regardless of patient‐ and disease‐related factors.

## 1 INTRODUCTION

Although psoriasis was originally regarded as primarily driven by Th1 cell‐derived cytokines, the current accepted pathogenetic mechanism highlights the central role of IL23/Th17 axis in psoriasis pathogenesis. IL17 and IL23 are now considered as key mediators in psoriasis inflammatory cascade.^1^, ^2^ In fact, the production of IL23 by dermal dendritic cells (DCs) drives IL17 expression, leading to inflammation, neutrophilic chemotaxis and uncontrolled keratinocytes proliferation.^3^ Based on these findings, several new biologic agents targeting IL23/Th17 axis have been developed for the treatment of plaque psoriasis.^4^ These new drugs include risankizumab, a humanized immunoglobulin G1 (IgG1) monoclonal antibody that selectively binds with high affinity to the p19 subunit of IL23. The efficacy and safety of risankizumab has been assessed in several phase III trials,^5^ in which risankizumab showed superior efficacy to both placebo and ustekinumab, with a favorable safety profile. In ULTIMMA‐1, at week 16, 75.3% of patients treated with risankizumab achieved a PASI90 response compared with 42% of patient receiving ustekinumab, similarly to the results obtained in ULTIMMA‐2 study. The efficacy and safety of risankizumab has been also assessed with “head‐to‐head” comparisons with other biologics, such as secukinumab in the IMMerge trial,^6^ showing superior efficacy and similar safety compared with secukinumab, plus a more favorable dosing regimen.

Despite randomized controlled trials (RCTs) are essential to assess the efficacy and safety of a certain drug, real‐life data often show differences between registration studies and clinical daily practice, since the latter displays a greater variability in demographical and clinical characteristics of treated patients in terms of age, disease severity, previous therapies and comorbidities. Thus, to integrate data from clinical trials with those of daily practice is essential to optimize patient's therapeutic management in a real‐world setting. To our knowledge, few real‐life studies have been performed to investigate the efficacy and safety of risankizumab, with an observation period ranging from 16 to 52 weeks.^7^, ^8^, ^9^, ^10^ The aim of this multicenter, retrospective study was to analyze the efficacy and safety of risankizumab in a population of patient from Lazio region in Italy affected by moderate‐to‐severe psoriasis, over a 52‐week treatment period.

## 2 MATERIALS AND METHODS

A retrospective analysis was performed in a cohort of patients with chronic plaque psoriasis, with or without psoriatic arthritis, who initiated treatment with risankizumab between July 2019 and December 2020. The study population consisted of patients attending the outpatient clinics of the nine participating centers (Fondazione Policlinico Universitario A. Gemelli IRCCS; IFO San Gallicano; Ospedale Sant'Andrea, Università di Roma La Sapienza – Polo Pontino; Università Campus Biomedico; Policlinico Umberto I; Ospedale Nuovo Regina Margherita; Istituto Dermopatico dell'Immacolata – IDI; Policlinico Tor Vergata; Ospedale di Belcolle, Viterbo) in Lazio, Italy. All enrolled patients were >18 years old and affected by chronic plaque psoriasis. Patients with generalized or palmoplantar pustular psoriasis, erythrodermic psoriasis or who had started treatment within a clinical trial were excluded, as well as patients concurrently treated with other systemic therapies. Risankizumab was administered at EMA approved dosage, and no dose or frequency variations were permitted. For each patient, demographic and clinical data (age, sex, weight, height, body mass index [BMI], age of onset and duration of psoriasis, family history, comorbidity), previous therapeutic history (phototherapy, cyclosporine, acitretin, methotrexate and biological therapies) and special‐site involvement (palmoplantar, scalp psoriasis, genital, facial areas) were collected at the time of initiation of biological therapy. In particular, data regarding the last biological treatment before starting risankizumab were collected, as well as the duration of the treatment and reasons of drug withdrawal. The severity of psoriasis was estimated by Psoriasis Area and Severity Index – PASI^11^ measured at baseline and after 4, 16, 28 and 52 weeks, respectively. Data about potential safety issues and adverse events (AEs) were collected, including mild and serious adverse events. The entire study was conducted according to the principles of the Helsinki Declaration.

### 2.1 Statistical analysis

Descriptive data were summarized using absolute and relative (%) frequencies for categorical variables, and medians and interquartile ranges (IR) for continuous non normally distributed variables. The percentual difference between the baseline PASI and the PASI score at different timing (4, 16, 28 and 52 weeks after the start of the treatment) was calculated to evaluate clinical responses set as PASI75, PASI90 and PASI100 (i.e., whether patients reached an improvement of 75%, 90% and 100% from baseline at weeks 4, 16, 28 and 52). Univariable and multivariable logistic regressions were used to understand variables that predicted the clinical response. In addition, we also used the “non‐responder imputation” analysis: conservative method that imputes that participants dropouts are considered non‐responders, regardless of actual response at the time of follow up loss.^12^ Statistical significance was set at *p*‐value <0.05. Analyses were performed by using STATA 13.0 Software (StataCorp, Texas).

## 3 RESULTS

We analyzed data regarding 112 patients with moderate‐to‐severe psoriasis that started biological therapy with risankizumab. The median age was 48 years (range 39–57) and 71 (63.39%) were males. More details regarding the demographic and clinical characteristics of the study population are described in Table 1.

**TABLE 1:** Clinical and demographic characteristics of the study population

Characteristics (total *n* = 112)	*N* (%); Median [IQR]
Gender	
Male	71 (63.39)
Female	41 (36.61)
Age	48 [39.50–57.00]
BMI	26.99 [24.27–29.47]
Arthropathy	
No	83 (74.11)
Yes	29 (25.89)
Familiary history	
No	54 (54.55)
Yes	45 (45.45)
Age of onset	24 [16.00–35.00]
Treatment duration (mo) with Risankizumab	15.23 [8.53–17.23]
Hand and foot psoriasis	
No	96 (87.27)
Yes	14 (12.73)
Genital psoriasis	
No	79 (71.82)
Yes	31 (28.18)
Scalp psoriasis	
No	41 (36.61)
Yes	71 (63.39)
Facial psoriasis	
No	72 (64.86)
Yes	39 (35.14)
Previous treatment	
Phototherapy	37 (33.64)
CyA	79 (71.17)
Methotrexate	61 (54.95)
Acitretin	29 (26.36)
Apremilast	8 (7.27)
Infliximab	9 (8.18)
Etanercept	35 (8.18)
Adalimumab	41 (36.94)
Golimumab	2 (1.82)
Certolizumab	1 (0.91)
Ustekinumab	21 (19.09)
Secukinumab	17 (15.60)
Ixekizumab	6 (5.45)
Guselkumab	7 (6.36)
Brodalumab	3 (2.68)
Last biological treatment	
Naive	47 (41.96)
Anti‐TNFα	35 (31.25)
Anti‐IL17	15 (13.39)
Anti‐IL23 or Anti‐IL12/23	15 (13.39)
Treatment suspension	
No	58 (85.29)
Yes	10 (14.71)
PASI at baseline	15.25 [10–20]
PASI at week 4	5.00 [2.00–8.00]
PASI at week 16	0.00 [0.00–2.00]
PASI at week 28	0.00 [0.00–0.20]
PASI at week 52	0.00 [0.00–0.00]

Abbreviations: BMI, Body Mass Index; CyA, cyclosporin A; PASI, Psoriasis Area and Severity Index.

**FIGURE 1:**
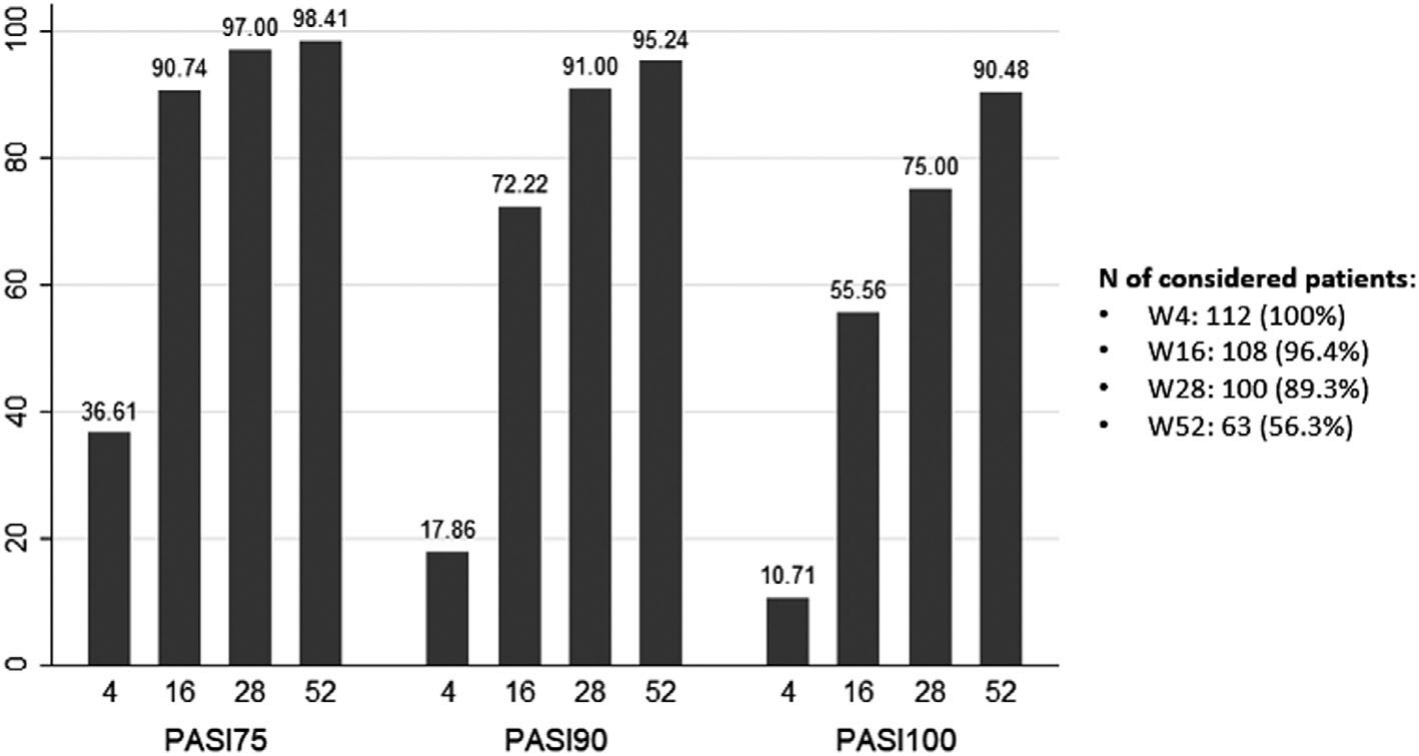
Proportions (%) of patients achieving PASI75, PASI90 and PASI100 responses at week 4, 16, 28 and 52

**FIGURE 2:**
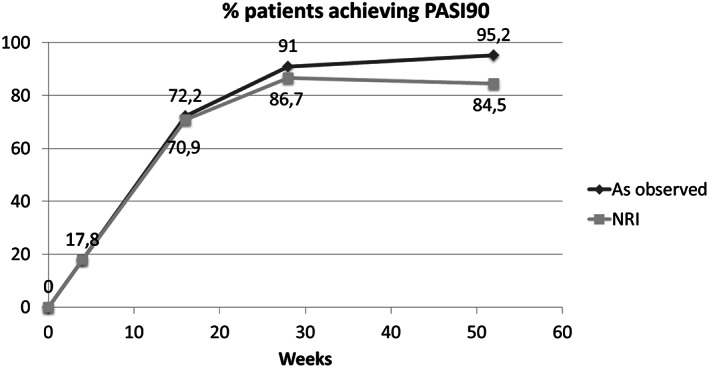
Proportions of PASI90 responders from baseline to week 52

Patients followed until weeks 4, 16, 28 and 52 were respectively: 112, 108, 100 and 63, with a treatment duration with risankizumab of 15.23 months (range 8.53–17.23) and a mean PASI score at baseline of 15.25 (range 10–20). Naïve and bio‐experienced patients showed similar mean PASIs at baseline (15.2 and 15.3, respectively). In our population, 41 (36.61%) patients achieved a PASI75 response at week 4, 20 (17.86%) achieved a PASI90, and 12 (10.71%) were clear of disease (PASI100). PASI scores continued to improve through to week 16, with 98 of 108 (90.74%) patients achieving PASI75, 78 (72.22%) patients achieving PASI90, and 60 (55.56%) patients achieving PASI100. At week 28, 97 out of 100 (97.0%) patients reached PASI75, 91 (91.0%) patients reached PASI90 and 75 (75.0%) patients reached PASI100. At week 52, data were available for 63 of 112 (56.25%) patients, with 98.41% (61 out of 63) of these patients achieving PASI75, 95.24% (60 out of 63) achieving PASI90 and 90.48% (57 out of 63) achieving PASI100 (Figure 1). Data retrieved from the NRI analysis about PASI75, PASI90 and PASI100 responses at the considered time‐points are represented in Figure S1. The proportions of patients reaching PASI90 response at the different time‐points are represented in Figure 2, where also data regarding the NRI analysis are depicted.

Univariate logistic regression analysis showed no substantial associations between the considered variables and the efficacy endpoints (PASI75, PASI90 and PASI100), only displaying sporadic, statistically significant associations not confirmed in the different time‐points and for each clinical endpoint). As for the PASI90, older age is positively associated with the odds of achieving clinical response only at week 4 (OR 1.05, *p* = 0.022), similarly to the presence of genital psoriasis (OR 6.50, *p* = 0.001) and a previous apremilast therapy (OR 5.80, *p* = 0.021). No significant associations were found between the analyzed variables and the odds of obtain a PASI90 response at week 16, while the presence of familiarity for psoriasis was negatively associated (OR 0.08, *p* = 0.019) to the achievement of PASI90 only at week 28. A similar trend, with no significant associations with PASI90 response, was observed at week 52, although with limitations due to the small number of patients followed up to 52 weeks. BMI had no impact on PASI75, PASI90 and PASI100 response rates even stratifying the study population in patients with BMI < 30 and BMI ≥ 30. Similarly, the history of previous biological therapies had no influence on the efficacy endpoints except for an isolate association between a previous therapy with an anti‐IL17 drug and lower odds of obtaining a PASI75 response at week 16 (OR 0.05, *p* = 0.008) Moreover, baseline PASI showed no influence on PASI90 response and only few sporadic associations with PASI75 and PASI100 were found at the different time‐points. Complete data regarding the univariate analysis on PASI90 response are reported in Table 2. A multivariate regression analysis (adjusted for age, sex, BMI, baseline PASI and the last biological drug used before starting risankizumab) showed no significant associations with PASI75, PASI90 and PASI100 responses at week 4, 16 and 28 except for sporadic associations for PASI90 response (see Table 3). Multivariate analysis at week 52 was not performed due to the small number of patients reaching this time‐point. Univariate and multivariate logistic regression analysis of variables influencing PASI90 response using the NRI analysis are represented in Tables S1 and S2, respectively, confirming no significant associations between the odds of obtaining a PASI90 response and the considered variables.

**TABLE 2:** Univariate logistic regression analysis of variables influencing PASI90 response at week 4, 16, 28 and 52

Variables	PASI 90 Week 4		PASI 90 Week 16		PASI 90 Week 28		PASI 90 Week 52	
	OR (95% CI)	*p*‐Value	OR (95% CI)	*p*‐Value	OR (95% CI)	*p*‐Value	OR (95% CI)	*p*‐Value
Age	**1.05 (1.01–1.09)**	**0.022**	1.00 (0.97–1.03)	0.990	0.96 (0.91–1.01)	0.183	0.91 (0.81–1.02)	0.116
Gender: female	Ref	0.500	Ref	0.602	Ref	0.629		^a^
Male	1.43 (0.50–4.07)		1.26		1.41 (0.36–5.60)			
BMI	1.04 (0.07–1.11)	0.318	1.01 (0.94–1.08)	0.792	0.98 (0.89–1.08)	0.631	0.96 (0.83–1.09)	0.516
BMI <30	Ref	0.056	Ref	0.056	Ref	0.056	Ref	0.056
≥30	2.74 (0.98–7.69)		2.74 (0.97–7.69)		2.74 (0.98–7.69)		2.74 (0.97–7.69)	
Arthropathy: No	Ref	0.920	Ref	0.609	Ref	0.840	Ref	0.800
Yes	0.94 (0.31–2.88)		1.29 (0.48–3.44)		1.18 (0.23–6.11)		0.72 (0.06–8.57)	
Family history: No	Ref	0.075	Ref	0.063	**Ref**	**0.019**	Ref	0.522
Yes	3.13 (0.89–10.94)		0.43 (0.17–1.05)		**0.08 (0.01–0.66)**		0.44 (0.04–5.23)	
Age of onset	1.01 (0.98–1.05)	0.389	0.99 (0.96–1.02)	0.554	0.99 (0.95–1.94)	0.761	0.95 (0.87–1.03)	0.185
Baseline PASI	0.94 (0.85–1.03)	0.174	1.03 (0.96–1.08)	0.432	1.09 (0.98–1.21)	0.131	1.20 (0.94–1.55)	0.137
Hand and foot psoriasis: No	Ref	0.752	Ref	0.511	Ref	0.074		^a^
Yes	0.77 (0.16–3.78)		0.67 (0.21–2.20)		0.25 (0.05–1.14)			
Genital psoriasis: No	**Ref**	**0.001**	Ref	0.232	Ref	0.259	Ref	0.852
Yes	**6.50 (2.25–18.76)**		0.58 (0.23–1.42)		0.45 (0.11–1.81)		0.79 (0.06–9.30)	
Scalp Psoriasis: No	Ref	0.098	Ref	0.201	Ref	0.763	Ref	0.381
Yes	2.60 (0.83–8.69)		1.75 (0.74–4.13)		0.80 (0.19–3.41)		3.00 (0.26–34.95)	
Facial Psoriasis: No	Ref	0.486	Ref	0.188	Ref	0.982	Ref	0.953
Yes	1.43 (0.52–3.92)		0.56 (0.23–1.33)		0.98 (0.23–4.21)		1.07 (0.09–12.58)	
Previous Phototherapy: No	Ref	0.745	Ref	0.589	Ref	0.982	Ref	0.862
Yes	1.19 (0.42–3.32)		1.29 (0.52–3.20)		0.98 (0.23–4.21)		1.24 (0.11–14.50)	
Previous Cyclosporin A: No	Ref	0.227	Ref	0.743	Ref	0.640	Ref	0.748
Yes	0.54 (0.20–1.47)		0.85 (0.33–2.20)		0.68 (0.13–3.47)		1.50 (0.13–17.74)	
Previous Methotrexate: No	Ref	0.141	Ref	0.939	Ref	0.978		^a^
Yes	2.18 (0.77–6.19)		0.97 (0.41–2.26)		1.02 (0.26–4.05)			
Previous Acitretin: No	Ref	0.572	Ref	0.652	Ref	0.736	Ref	0.748
Yes	1.36 (0.46–4.00)		1.25 (0.47–3.36)		1.33 (0.26–6.82)		0.67 (0.56–7.89)	
Previous Apremilast: No	**Ref**	**0.021**	Ref	0.551	Ref	0.123	Ref	0.193
Yes	**5.80 (1.31–25.74)**		0.63 (0.14–2.84)		0.25 (0.04–1.46)		0.18 (0.01–2.37)	
Last biological drug: Naive	Ref		Ref		Ref			
Anti‐TNF	0.48 (0.14–1.67)	0.248	0.71 (0.25–1.96)	0.504		^a^		^a^
Anti‐IL17	1.35 (0.35–5.14)	0.664	0.34 (0.10–1.16)	0.084		^a^		^a^
Anti‐IL23	0.57 (0.11–2.95)	0.502	1.18 (0.28–5.01)	0.826		^a^		^a^

*Note*: In bold characters, statistically significant associations.

Abbreviations: BMI, Body Mass Index; PASI, Psoriasis Area and Severity Index.

^a^ Too few non‐responders.

**TABLE 3:** Multivariate logistic regression analysis of variables influencing PASI90 response at week 4, 16 and 28

Variables	PASI 90 Week 4		PASI 90 Week 16		PASI 90 Week 28	
	OR (95% CI)	*p*‐Value	OR (95% CI)	*p*‐Value	OR (95% CI)	*p*‐Value
Age	**1.04 (1.00–1.08)**	**0.046**	1.00 (0.96–1.03)	0.912	0.97 (0.91–1,03)	0.266
Gender: female	Ref	0.877	Ref	0.637	Ref	0.440
Male	1.09 (0.35–3.36)		1.24 (0.50–3.11)		1.78 (0.41–7.78)	
BMI	1.04 (0.96–1.14)	0.346	1.00 (0.93–1.08)	0.910	0.96 (0.85–1.08)	0.526
Baseline PASI	**0.93 (0.86–0.99)**	**0.045**	1.02 (0.97–1.08)	0.476	1.07 (0.97–1.20)	0.166
Bio‐naïve	Ref	0.308	Ref	0.353	Ref	0.715
Bio‐experienced	0.58 (0.21–1.64)		0.66 (0.30–1.60)		1.31 (0.31–5.57)	

Abbreviations: BMI, Body Mass Index; PASI, Psoriasis Area and Severity Index.

Significance of bold values is *p*‐value < 0.05

Treatment suspension was recorded in 10 (14.71%) patients; 6 patients were lost to follow up, 2 patients stopped treatment because of COVID‐19 pandemic‐related logistical difficulties, 1 patient for a planned election surgery and 1 patient for worsening of psoriatic arthritis together with a minimal worsening of a pre‐existing ulcerative colitis. No mild or serious safety issues occurred during the observation period, and no discontinuations related to AEs were reported.

## 4 DISCUSSION

This multicenter, retrospective study analyzed a cohort of patients from Lazio region with moderate to severe psoriasis, treated with risankizumab with a maximum follow‐up period of 52 weeks. The results of our study confirm the efficacy and safety of risankizumab in psoriatic patients in line with the findings of RCTs, with significant reduction of PASI scores at the considered time‐points without reported safety issues. In our population a significant proportion of patients (72.22%) achieved a PASI90 response at week 16, increasing to 95.24% at week 52 for the patients reaching this time‐point. The results at week 16 are comparable to those reported in RCTs, in which 75.3% of the patients in the UltIMMA‐1 trial and 74.8% in the UltIMMA‐2 trial reached a PASI90 response after 4 months of therapy, while the proportion of patients achieving a PASI90 response at week 52 was found slightly higher in our study respect to data reported in UltIMMA‐1 and ‐2 trials (81.9% and 80.6%, respectively). A possible explanation of this discrepancy can be found in the small sample size of our study compared to those of RCTs. Analogous findings are available from other real‐life experiences with risankizumab. However, as previously stated, few studies concerning the efficacy and safety of risankizumab in a real‐world setting are currently available, mostly with a short period of observation or small sample sizes. Megna et al.^7^, ^13^ results highlighted the efficacy and safety of risankizumab in two 16‐week retrospective studies even in psoriatic patients who previously failed a biologic therapy with other molecules, showing a decrease of mean PASI score from baseline (11.9) to week 16 (3.3). Another real‐life multicenter study carried out during COVID‐19 pandemic in a cohort of 57 psoriatic patients treated with risankizumab and observed up to 16 weeks further confirmed the efficacy and safety of this drug, with 63.2% of patients reaching a PASI90 response at week 16 and with no reported SARS‐CoV‐2 infections.^10^ The same Authors later confirmed these results in an extension analysis in the same cohort of patients up to 52 weeks of observation.^14^ Lastly, Ruggiero et al^15^ performed an indirect real‐life comparison between guselkumab‐ and risankizumab‐treated patients, recording similar profiles in terms of efficacy and safety over 44 weeks of observation. To our knowledge, the only real‐life study concerning risankizumab and providing a long period of observation along with a significant number of patients is a recent multicenter study from Czech Republic that analyzed a cohort of 154 patients undergoing risankizumab therapy with a follow up period of 52 weeks.^9^ The authors found that 63.8% of the patients included achieved a PASI90 response at week 16, increasing up to 82.4% at week 52.

In our study, the univariate and multivariate analysis carried out to assess the influence of patient‐ and disease‐related factors also showed similar results to those reported from clinical trials. In UltIMMa‐2 trial,^16^ a sub‐analysis was conducted to analyze the factors influencing the achievement of PASI90 response with risankizumab at week 16 and 52 (age, sex, BMI category, baseline PASI, baseline PGA, PsA and history of previous biological therapies) and the authors found that the response was not affected by any of the above‐mentioned variables. Similarly, our results showed that the efficacy of risankizumab in a real‐world setting does not seem to be influenced by the variables considered in our analysis, providing a rapid and sustained improvement of skin lesions regardless of demographic and clinical characteristics of the patient. For example, we found that BMI did not affect short‐ and long‐term effectiveness of risankizumab in our patients. Interestingly, this is in line with a previous study by Gkalpakiotis et al.^9^ in which no differences were detected in the efficacy of risankizumab comparing patients with BMI < 25 and BMI ≥ 25 up to 52 weeks of observation. In contrast with our findings and with those reported in the Czech study, Hansel et al. highlighted how BMI > 25 could negatively affect the efficacy of risankizumab^10^ similarly to the retrospective study of Borroni et al. in which increasing body mass index decreased the chances of reaching PASI 90 at week 40,^17^ even though the short period of observation and the small sample size could represent a limitation.

In our opinion, our findings are worthy of note because various studies have shown that several categories of patients, such as obese, bio‐experienced and those with high PASI at baseline, have a very low probability to achieve a consistent clinical improvement with numerous molecules, in particular with anti TNF agents.^18^, ^19^, ^20^, ^21^, ^22^, ^23^, ^24^, ^25^ These are the first class of biologics approved for the treatment of moderate‐to‐severe chronic plaque psoriasis and, in the last years, after the approval of their biosimilars, they are often recommended as first‐line treatment of choice for psoriatic patients, based upon economic reasons. Then, taking into account our data that have confirmed the effectiveness of risankizumab in all categories of patients, we suggest that starting with this drug or with other non‐antiTNF drugs with similar real‐life data might be advisable in those “high‐need” categories of patients who have a high probability to be unresponsive to anti‐TNF agents. This might help to individualize biologic therapy, improving outcomes and patient satisfaction and really rationalizing costs.

Lastly, our study provided an observation period up to 52 weeks, in which risankizumab has been shown to ensure a sustained clinical response over time, also displaying a favorable safety profile. These results are in line with the findings reported in an interim analysis of the LIMMitless open‐label extension trial,^26^ where risankizumab showed high‐efficacy rates with a maintenance of clinical response over 172 weeks. Literature data show that long persistence on treatment seems the strength of anti‐IL23 drugs, against the potential long‐term loss of efficacy shown by other molecules.^27^ A recent retrospective study compared the drug survival of anti‐IL12/23, ‐IL17 and ‐IL23 drugs in a large cohort of psoriatic patients,^28^ highlighting better long‐term performances for anti‐IL23 drugs (guselkumab and risankizumab) as opposed to the worse drug survival of secukinumab which was also inferior to ustekinumab considered as the reference drug. One possible explanation for this difference between anti‐IL23 and anti‐IL17 drugs may be the impact of inhibition of IL23 action on immune memory. In fact, tissue‐resident memory cells seem to be implicated in the pathogenesis of psoriasis and in potential disease recurrences, as opposed to regulatory T cells that instead play an inhibitory role. Recent evidence shows that IL23 plays a role in promoting the pathogenetic role of tissue‐resident memory cells.^29^ In a study comparing changes in the immunologic profile during therapy with guselkumab and secukinumab, the former was proven to reduce the number of tissue‐resident memory cells in favor of regulatory T cells, while, in the secukinumab group, a decrease in regulatory T cells was found.^30^

The limitations of this study are mainly represented by the retrospective method of data collection and the absence of a control group. Moreover, the relatively small sample size and the smaller proportion of subjects reaching 52 weeks of treatment respect to the entire study population could result in a less accurate analysis of long‐term effectiveness.

In conclusion, despite the introduction of biological therapies in psoriasis' therapeutic ladder dramatically improved the treatment of moderate‐to‐severe forms of this disease, the influence of several patient‐ and disease‐related factors can impair the efficacy of these drugs, stressing the need for treatments which demonstrate efficacy and safety regardless of external variables. In this context, in our study risankizumab showed high effectiveness maintained over time together with a favorable safety profile, without being influenced from variables such as BMI, prior failures with other biological therapies and baseline disease severity. These real‐life data results confirm the findings of RCTs and emphasize the possibility of achieving high‐efficacy rates with risankizumab even in patient groups with unfavorable characteristics.

## CONFLICT OF INTEREST

The authors declare no conflict of interest.

## Supporting information


**FIGURE S1**: Proportions (%) of patients achieving PASI75, PASI90 and PASI100 responses at week 4, 16, 28 and 52 according to NRI analysis.

**TABLE S1**: Univariate logistic regression analysis of variables influencing PASI90 response at week 4, 16, 28 and 52 using the non‐responder imputation analysis. In bold characters, statistically significant associations.
**TABLE S2**: Multivariate logistic regression analysis of variables influencing PASI90 response at week 4, 16 and 28 using the non‐responder imputation analysis.

## Data Availability

The data that support the findings of this study are available from the corresponding author upon reasonable request.
