# Supplementary figures and images for: Risankizumab for the treatment of moderate‐to‐severe psoriasis: A multicenter, retrospective, 1 year real‐life study
Source: Dermatol Ther. 2022 Apr 13;35(6):e15489. doi: 10.1111/dth.15489 (PMC9287038; doi:10.1111/dth.15489)

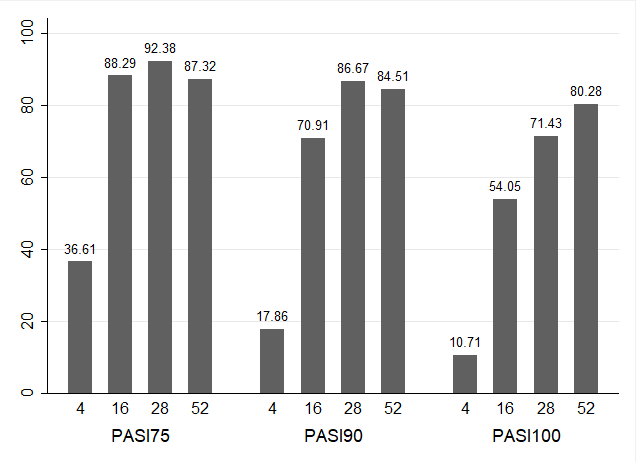

Supplement: Supplementary file 1 — FIGURE S1: Proportions (%) of patients achieving PASI75, PASI90 and PASI100 responses at week 4, 16, 28 and 52 according to NRI analysis. [file DTH-35-0-s001.png]
