# Supplementary material for: Risankizumab for the treatment of moderate‐to‐severe psoriasis: A multicenter, retrospective, 1 year real‐life study
Source: Dermatol Ther. 2022 Apr 13;35(6):e15489. doi: 10.1111/dth.15489 (PMC9287038; doi:10.1111/dth.15489)
Supplement: Supplementary file 2 — TABLE S1: Univariate logistic regression analysis of variables influencing PASI90 response at week 4, 16, 28 and 52 using the non‐responder imputation analysis. In bold characters, statistically significant associations. TABLE S2: Multivariate logistic regression analysis of variables influencing PASI90 response at week 4, 16 and 28 using the non‐responder imputation analysis. [file DTH-35-0-s002.docx]

**SUPPLEMENTARY TABLES (DTH-21-2212)**

**Supplementary Table 1 – Univariate logistic regression analysis of variables influencing PASI90 response at week 4,16, 28 and 52 using the Non-Responder Imputation Analysis. In bold characters, statistically significant associations**

| **Variables** | **PASI 90 Week 4** | | **PASI 90 Week 16** | | **PASI 90 Week 28** | | **PASI 90 Week 52** | |
| --- | --- | --- | --- | --- | --- | --- | --- | --- |
|  | **OR (95%CI)** | ***P*-value** | **OR (95%CI)** | ***P*-value** | **OR (95%CI)** | ***P*-value** | **OR (95%CI)** | ***P*-value** |
| Age | **1.05 (1.00 – 1.09)** | **0.022** | 1.01 (0.98 – 1.04) | 0.692 | 0.99 (0.95 – 1.04) | 0.791 | 1.00 (0.95 – 1.05) | 0.999 |
| Gender: female | Ref | 0.500 | Ref | 0.369 | Ref | 0.635 | Ref | 0.375 |
| male | 1.43 (0.50 – 4.07) |  | 1.47 (0.63 – 3.40) |  | 1.32 (0.42 – 4.13) |  | 0.53 (0.13 – 2.18) |  |
| BMI | 1.04 (0.96 – 1.11) | 0.318 | 1.01 (0.94 – 1.08) | 0.782 | 0.98 (0.90 – 1.06) | 0.576 | 0.97 (0.89 -1.06) | 0.509 |
| BMI <30 | Ref | 0.056 | Ref | 0.056 | Ref | 0.056 | Ref | 0.056 |
| ≥30 | 2.74 (0.98 – 7.69) |  | 2.74 (0.97 – 7.69) |  | 2.74 (0.98 – 7.69) |  | 2.74 (0.98 -7.69) |  |
| Arthropathy: No | Ref | 0.920 | Ref | 0.495 | Ref | 0.310 | Ref | 0.967 |
| Yes | 0.94 (0.31 – 2.88) |  | 1.40 (0.53 – 3.71) |  | 1.24 (0.31 – 4.84) |  | 0.97 (0.23 – 4.11) |  |
| Familiarity: No | Ref | 0.075 | Ref | 0.066 | **Ref** | **0.011** | Ref | 0.426 |
| Yes | 3.13 (0.89 – 10.94) |  | 0.44 (0.18 – 1.06) |  | **0.13 (0.03 – 0.61)** |  | 0.54 (0.12 – 2.47) |  |
| Age of onset | 1.01 (0.98 – 1.05) | 0.389 | 1.00 (0.97 – 1.02) | 0.846 | 1.01 (0.97 – 1.05) | 0.636 | 1.01 (0.96 – 1.06) | 0.761 |
| Baseline PASI | 0.93 (0.87 – 1.00) | 0.059 | 1.01 (0.96 – 1.06) | 0.632 | 1.08 (0.99 – 1.17) | 0.085 | **1.14 (1.02 – 1.28)** | **0.025** |
| Hand and foot psoriasis: No | Ref | 0.752 | Ref | 0.594 | Ref | 0.084 | Ref | 0.699 |
| Yes | 0.77 (1.56 – 3.78) |  | 0.73 (0.22 – 2.36) |  | 0.31 (0.08 – 1.71) |  | 1.54 (0.17 – 13.69) |  |
| Genital psoriasis: No | **Ref** | **0.001** | Ref | 0.322 | Ref | 0.469 | Ref | 0.943 |
| Yes | **6.50 (2.25 – 18.76)** |  | 0.64 (0.26 – 1.56) |  | 0.64 (0.20 – 2.11) |  | 1.05 (0.25 – 4.45) |  |
| Scalp Psoriasis: No | Ref | 0.98 | Ref | 0.304 | Ref | 0.478 | Ref | 0.428 |
| Yes | 2.69 (0.83 – 8.68) |  | 1.56 (0.67 – 3.61) |  | 0.64 (1.19 – 2.10) |  | 0.56 (0.13 – 2.34) |  |
| Facial Psoriasis: No | Ref | 0.486 | Ref | 0.903 | Ref | 0.470 | Ref | 0.227 |
| Yes | 1.43 (0.52 – 3.92) |  | 0.48 (0.21 – 1.13) |  | 0.66 (0.21 – 2.06) |  | 0.45 (0.12 – 1.65) |  |
| Previous Phototherapy: No | Ref | 0.745 | Ref | 0.457 | Ref | 0.744 | Ref | 0.487 |
| Yes | 1.19 (0.42 – 3.32) |  | 1.41 (0.57 – 3.47) |  | 1.23 (0.36 – 4.25) |  | 1.66 (0.40 – 6.89) |  |
| Previous Cyclosporin A: No | Ref | 0.227 | Ref | 0.855 | Ref | 0.528 | Ref | 0.628 |
| Yes | 0.54 (0.20 – 1.47) |  | 0.92 (0.37 – 2.29) |  | 0.64 (0.17 – 2.50) |  | 0.67 (0.13 – 3.35) |  |
| Previous Methotrexate: No | Ref | 0.141 | Ref | 0.795 | Ref | 0.673 | Ref | 0.743 |
| Yes | 2.18 (0.77 – 6.19) |  | 1.12 (0.49 – 2.56) |  | 1.28 (0.41 – 3.93) |  | 0.80 (0.21 – 3.03) |  |
| Previous Acitretin: No | Ref | 0.572 | Ref | 0.534 | Ref | 0.932 | Ref | 0.175 |
| Yes | 1.36 (0.46 – 4.00) |  | 1.36 (0.51 – 3.62) |  | 0.95 (0.27 – 3.30) |  | 0.40 (0.11 – 1.50) |  |
| Previous Apremilast: No | **Ref** | **0.021** | Ref | 0.614 | Ref | 0.325 | Ref | 0.934 |
| Yes | **5.8 (1.30 – 25.74)** |  | 0.68 (1.15 – 3.03) |  | 0.43 (0.08 – 2.34) |  | 0.91 (0.10 – 8.63) |  |
| Last biological drug: Naive | Ref |  | Ref |  | Ref |  | Ref |  |
| Anti-TNF | 0.48 |  | 0.85 (0.32 – 2.28) | 0.742 | 1.94 (0.46 – 8.18) | 0.364 | 1.53 (0.33 – 7.15) | 0.591 |
| Anti-IL17 | 1.35 |  | 0.40 (0.12 – 1.35) | 0.141 | 0.71 (1.16 – 3.23) | 0.661 | 0.83 (0.13 – 5.16) | 0.845 |
| Anti-IL23 | 0.57 |  | 1.41 (0.34 – 5.88) | 0.636 | 2.72 (0.31 – 24.19) | 0.369 | 1.25 (0.12 – 12.80) | 0.851 |

**Supplementary Table 2 - Multivariate logistic regression analysis of variables influencing PASI90 response at week 4, 16 and 28 using the Non-Responder Imputation Analysis**

| **Variables** | **PASI 90 Week 4** | | **PASI 90 Week 16** | | **PASI 90 Week 28** | |
| --- | --- | --- | --- | --- | --- | --- |
|  | **OR (95%CI)** | ***P*-value** | **OR (95%CI)** | ***P*-value** | **OR (95%CI)** | ***P*-value** |
| Age | **1.04 (1.00 – 1.09)** | **0.046** | 1.00 (0.97 – 1.05) | 0.818 | 1.00 (0.97 – 1.05) | 0.966 |
| Gender: female | Ref | 0.877 | Ref | 0.418 |  | 0.461 |
| male | 1.09 (0.35 – 3.36) |  | 1.44 (0.60 – 3.49) |  | 1.58 (0.47 – 5.38) |  |
| BMI | 1.04 (0.96 – 1.13) | 0.346 | 1.00 (0.93 – 1.07) | 0.977 | 0.94 (0.86 – 1.04) | 0.225 |
| Baseline PASI | 0.93 (0.86 – 0.99) | 0.045 | 1.01 (0.96 – 1.07) | 0.636 | 1.09 (0.99 –1.19) | 0.060 |
| Bio-naïve | Ref | 0.308 | Ref | 0.574 | Ref | 0.323 |
| Bio-experienced | 0.58 (0.21 – 1.64) |  | 1.59 (0.33 – 1.84) |  | 1.82 (0.56 – 5.95) |  |

BMI: Body Mass Index; PASI: Psoriasis Area and Severity Index
